# Supplementary material for: AR alterations inform circulating tumor DNA detection in metastatic castration resistant prostate cancer patients
Source: Nat Commun. 2024 Dec 11;15:10648. doi: 10.1038/s41467-024-54847-1 (PMC11634963; doi:10.1038/s41467-024-54847-1)
Supplement: Supplementary file 1 — Supplementary Information [file 41467_2024_54847_MOESM1_ESM.pdf]

Supplementary Table 1. Clinical characteristics of A031201 patients with and without cfDNA-seq data available.

	cfDNA-seq data (N=776)	No cfDNA-seq data (N=535)	Total (N=1311)
**Age**			
Median (Q1, Q3)	71.0 (65.0, 78.0)	72.0 (66.0, 78.0)	71.0 (65.0, 78.0)
**PSA_log**			
Median (Q1, Q3)	3.12 (2.22, 4.19)	3.26 (2.30, 4.32)	3.17 (2.25, 4.25)
Missing	0 (0%)	1 (0.2%)	1 (0.1%)
**HGB**			
Median (Q1, Q3)	12.9 (12.0, 13.7)	13.0 (12.1, 13.9)	13.0 (12.0, 13.8)
**ALB**			
Median (Q1, Q3)	4.00 (3.80, 4.30)	4.10 (3.80, 4.30)	4.00 (3.80, 4.30)
Missing	2 (0.3%)	1 (0.2%)	3 (0.2%)
**AlkPhos_log**			
Median (Q1, Q3)	4.49 (4.26, 4.87)	4.53 (4.22, 4.88)	4.50 (4.25, 4.88)
Missing	1 (0.1%)	3 (0.6%)	4 (0.3%)
**ANC**			
Median (Q1, Q3)	4.10 (3.20, 5.10)	3.71 (2.97, 4.73)	3.90 (3.10, 4.90)
Missing	2 (0.3%)	3 (0.6%)	5 (0.4%)
**LDH_log**			
Median (Q1, Q3)	5.28 (5.11, 5.49)	5.27 (5.13, 5.48)	5.27 (5.12, 5.48)
Missing	24 (3.1%)	22 (4.1%)	46 (3.5%)
**riskscore**			
Median (Q1, Q3)	-0.361 (-0.574, -0.0968)	-0.339 (-0.563, -0.122)	-0.355 (-0.571, -0.103)
Missing	3 (0.4%)	4 (0.7%)	7 (0.5%)
**Bone**			
No	142 (18.3%)	85 (15.9%)	227 (17.3%)
Yes	634 (81.7%)	450 (84.1%)	1084 (82.7%)
**Liver**			
No	745 (96.0%)	510 (95.3%)	1255 (95.7%)
Yes	31 (4.0%)	25 (4.7%)	56 (4.3%)
**Lung**			
No	690 (88.9%)	470 (87.9%)	1160 (88.5%)
Yes	86 (11.1%)	65 (12.1%)	151 (11.5%)
**Nodal**			
No	394 (50.8%)	287 (53.6%)	681 (51.9%)
Yes	382 (49.2%)	248 (46.4%)	630 (48.1%)
**Other_metastasis**			
No	710 (91.5%)	483 (90.3%)	1193 (91.0%)
Yes	66 (8.5%)	52 (9.7%)	118 (9.0%)
**Race**			
Black	86 (11.1%)	76 (14.2%)	162 (12.4%)
Other	35 (4.5%)	26 (4.9%)	61 (4.7%)
White	655 (84.4%)	433 (80.9%)	1088 (83.0%)

Supplementary Table 1 (continued)

**Ethnicity**			
Hispanic or Latino	29 (3.7%)	31 (5.8%)	60 (4.6%)
Not Hispanic or Latino	720 (92.8%)	486 (90.8%)	1206 (92.0%)
Not reported: Patient refused or data not available	15 (1.9%)	10 (1.9%)	25 (1.9%)
Unknown: Patient is unsure of their ethnicity	12 (1.5%)	8 (1.5%)	20 (1.5%)
**Prior_Chemotherapy**			
No	759 (97.8%)	525 (98.1%)	1284 (97.9%)
Yes	17 (2.2%)	10 (1.9%)	27 (2.1%)
**Halabi_Risk_Factor**			
High	124 (16.0%)	81 (15.1%)	205 (15.6%)
Intermediate	267 (34.4%)	196 (36.6%)	463 (35.3%)
Low	376 (48.5%)	255 (47.7%)	631 (48.1%)
Missing	9 (1.2%)	3 (0.6%)	12 (0.9%)
**Prior_Tumor**			
No	86 (11.1%)	47 (8.8%)	133 (10.1%)
Yes	690 (88.9%)	488 (91.2%)	1178 (89.9%)
**Gleason_Score**			
Greater than or equal to eight	442 (57.0%)	285 (53.3%)	727 (55.5%)
Less than or equal to six	77 (9.9%)	45 (8.4%)	122 (9.3%)
Missing	62 (8.0%)	56 (10.5%)	118 (9.0%)
Seven	195 (25.1%)	149 (27.9%)	344 (26.2%)
**PS**			
0	457 (58.9%)	306 (57.2%)	763 (58.2%)
1	319 (41.1%)	229 (42.8%)	548 (41.8%)
**Opioid**			
0	647 (83.4%)	434 (81.1%)	1081 (82.5%)
1	129 (16.6%)	101 (18.9%)	230 (17.5%)
**BSL**			
No	461 (59.4%)	320 (59.8%)	781 (59.6%)
Yes	315 (40.6%)	215 (40.2%)	530 (40.4%)
**LDH_ULN**			
0	599 (77.2%)	411 (76.8%)	1010 (77.0%)
1	177 (22.8%)	124 (23.2%)	301 (23.0%)

PSA, prostate specific antigen; HGB, hemoglobin; ALB, albumin; AlkPhos, alkaline phosphatase; ANC, absolute neutrophil count; LDH, lactate dehydrogenase; PS, performance status; ULN, upper limit of normal. ‘N’ refers to the number of patients.

Supplementary Table 2. Clinical characteristics of A031201 patients based on ctDNA group status.

	group1 (N=200)	group2 (N=256)	group3 (N=320)	P-value Group 1 vs. 3	P-value Group 2 vs. 3
**Age**					
Median (Q1, Q3)	70.0 (65.0, 76.0)	71.0 (64.0, 78.3)	71.0 (66.0, 77.0)		
**PSA_log**					
Median (Q1, Q3)	3.44 (2.50, 4.57)	3.26 (2.29, 4.39)	2.77 (1.85, 3.72)		
**HGB**					
Median (Q1, Q3)	12.6 (11.7, 13.5)	12.7 (11.7, 13.7)	13.2 (12.3, 13.9)		
**ALB**					
Median (Q1, Q3)	4.00 (3.70, 4.30)	4.00 (3.80, 4.30)	4.10 (3.90, 4.30)		
Missing	1 (0.5%)	0 (0%)	1 (0.3%)		
**AlkPhos_log**					
Median (Q1, Q3)	4.70 (4.37, 5.30)	4.52 (4.28, 5.03)	4.38 (4.20, 4.66)		
Missing	0 (0%)	1 (0.4%)	0 (0%)		
**ANC**					
Median (Q1, Q3)	4.13 (3.26, 5.30)	4.10 (3.25, 5.00)	3.95 (3.10, 4.92)		
Missing	1 (0.5%)	0 (0%)	1 (0.3%)		
**LDH_log**					
Median (Q1, Q3)	5.37 (5.19, 5.65)	5.27 (5.12, 5.48)	5.25 (5.06, 5.42)		
Missing	10 (5.0%)	5 (2.0%)	9 (2.8%)		
**riskscore**					
Median (Q1, Q3)	-0.204 (-0.499, 0.0367)	-0.300 (-0.520, -0.0565)	-0.434 (-0.637, -0.244)	< 0.001	< 0.001
Missing	1 (0.5%)	1 (0.4%)	1 (0.3%)		
**Bone**					
No	32 (16.0%)	39 (15.2%)	71 (22.2%)		
Yes	168 (84.0%)	217 (84.8%)	249 (77.8%)		
**Liver**					
No	189 (94.5%)	243 (94.9%)	313 (97.8%)		
Yes	11 (5.5%)	13 (5.1%)	7 (2.2%)		
**Lung**					
No	175 (87.5%)	229 (89.5%)	286 (89.4%)		
Yes	25 (12.5%)	27 (10.5%)	34 (10.6%)		
**Nodal**					
No	98 (49.0%)	132 (51.6%)	164 (51.3%)		
Yes	102 (51.0%)	124 (48.4%)	156 (48.8%)		
**Other_metastasis**					
No	181 (90.5%)	228 (89.1%)	301 (94.1%)		
Yes	19 (9.5%)	28 (10.9%)	19 (5.9%)		
**Race**					
Black	27 (13.5%)	34 (13.3%)	25 (7.8%)		
Other	13 (6.5%)	7 (2.7%)	15 (4.7%)		
White	160 (80.0%)	215 (84.0%)	280 (87.5%)		
**Ethnicity**					
Hispanic or Latino	7 (3.5%)	8 (3.1%)	14 (4.4%)		
Not Hispanic or Latino	186 (93.0%)	237 (92.6%)	297 (92.8%)		
Not reported: Patient refused or data not available	4 (2.0%)	5 (2.0%)	6 (1.9%)		
Unknown: Patient is unsure of their ethnicity	3 (1.5%)	6 (2.3%)	3 (0.9%)		
**Prior_Chemotherapy**					
No	195 (97.5%)	250 (97.7%)	314 (98.1%)		
Yes	5 (2.5%)	6 (2.3%)	6 (1.9%)		
**Halabi_Risk_Factor**					
High	47 (23.5%)	52 (20.3%)	25 (7.8%)	< 0.001	< 0.001
Intermediate	74 (37.0%)	95 (37.1%)	98 (30.6%)		
Low	74 (37.0%)	107 (41.8%)	195 (60.9%)		
Missing	5 (2.5%)	2 (0.8%)	2 (0.6%)		
**Prior_Tumor**					
No	24 (12.0%)	27 (10.5%)	35 (10.9%)		
Yes	176 (88.0%)	229 (89.5%)	285 (89.1%)		
**Gleason_Score**					
Greater than or equal to eight	127 (63.5%)	148 (57.8%)	167 (52.2%)		
Less than or equal to six	15 (7.5%)	24 (9.4%)	38 (11.9%)		
Seven	47 (23.5%)	61 (23.8%)	87 (27.2%)		
Missing	11 (5.5%)	23 (9.0%)	28 (8.8%)		

Supplementary Table 2 (continued)

**PS**			
0	118 (59.0%)	135 (52.7%)	204 (63.8%)
1	82 (41.0%)	121 (47.3%)	116 (36.3%)
**Opioid**			
0	159 (79.5%)	208 (81.3%)	280 (87.5%)
1	41 (20.5%)	48 (18.8%)	40 (12.5%)
**BSL**			
No	111 (55.5%)	149 (58.2%)	201 (62.8%)
Yes	89 (44.5%)	107 (41.8%)	119 (37.2%)
**LDH_ULN**			
0	133 (66.5%)	195 (76.2%)	271 (84.7%)
1	67 (33.5%)	61 (23.8%)	49 (15.3%)

PSA, prostate specific antigen; HGB, hemoglobin; ALB, albumin; AlkPhos, alkaline phosphatase; ANC, absolute neutrophil count; LDH, lactate dehydrogenase; PS, performance status; ULN, upper limit of normal. ‘N’ refers to the number of patients.

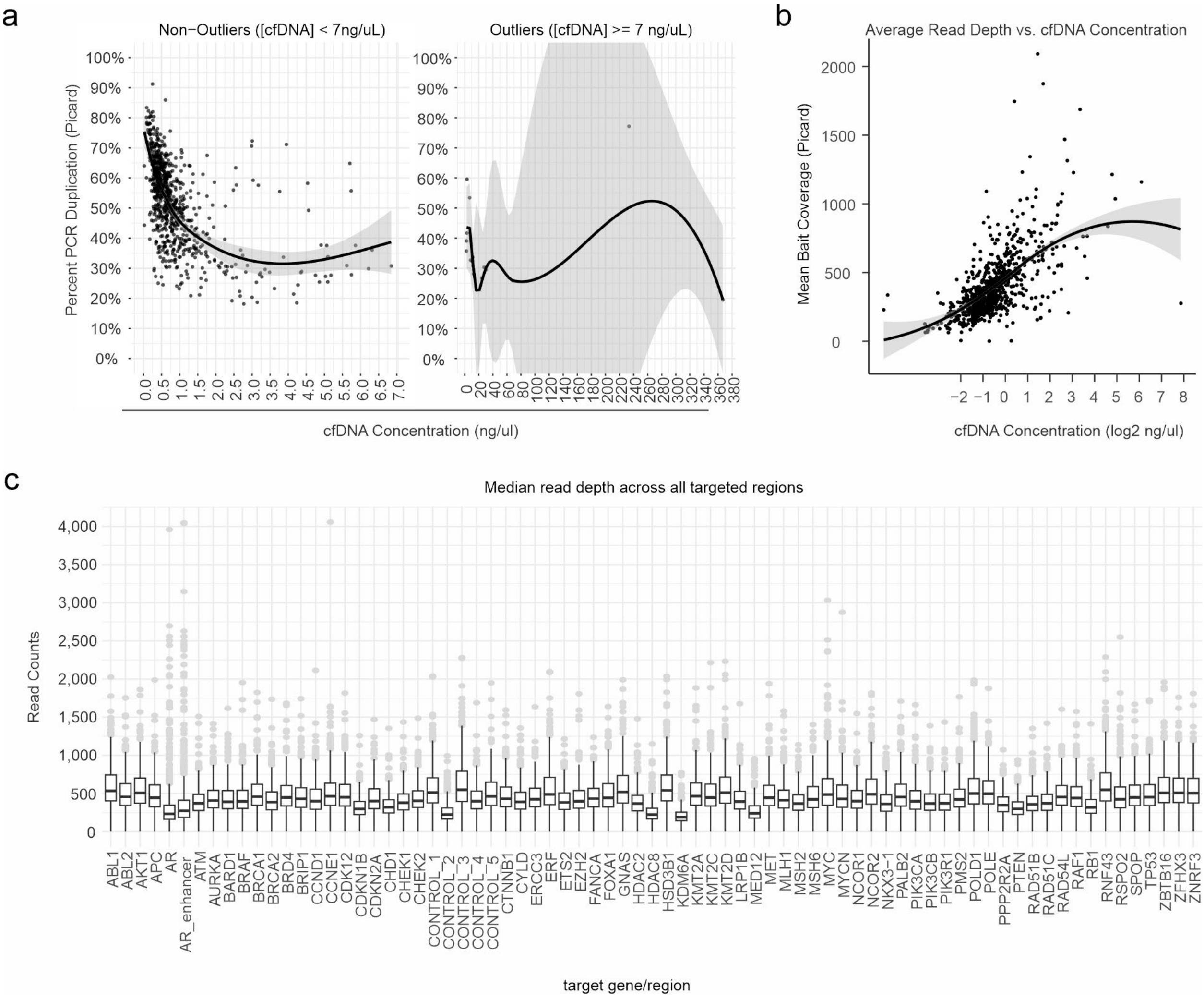

Supplementary Figure 1. DNA-sequencing metrics across 776 cfDNA specimens. (a) Scatterplot of percent PCR duplication vs. concentration of cfDNA used as input for DNA-seq library preparation across 776 cfDNA specimens. Loess trendline and 95% confidence interval are illustrated. **(b)** Scatterplot of average unique DNA-seq read coverage for all baits on the targeted DNA-seq panel vs. concentration of cfDNA used as input for DNA-seq library preparation across all 776 cfDNA specimens. Loess trendline and 95% confidence interval are illustrated. **(c)** Depth of unique DNA-seq reads for targeted genes and genomic regions across 776 cfDNA specimens. Boxes represent median and interquartile range. Whiskers represent 1.5X interquartile range. Outliers are illustrated as gray points.

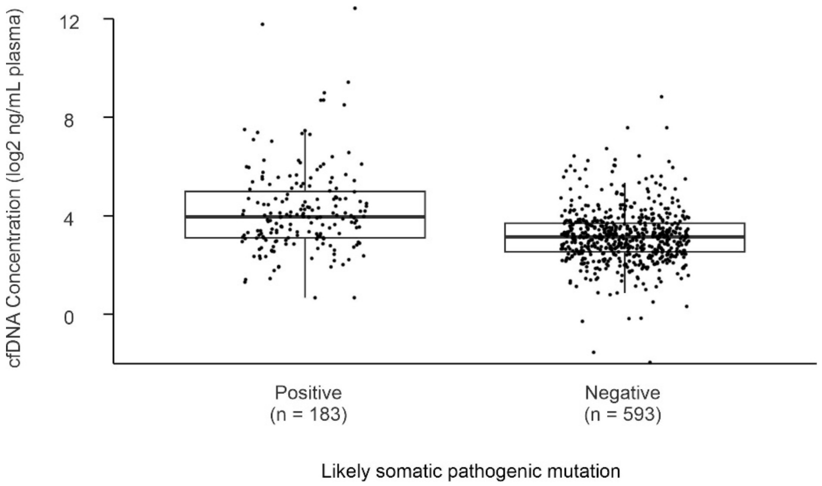

Supplementary Figure 2. cfDNA yields in cfDNA specimens harboring or lacking likely somatic pathogenic mutations. Boxplot of cfDNA yields (ng of cfDNA isolated per mL of plasma) for samples stratified by whether they harbor a likely somatic pathogenic mutation (n = 183) or lack detection of a likely somatic pathogenic mutation (n = 593). Boxes represent median and interquartile range. Whiskers represent 1.5X interquartile range. ‘n’ refers to the number of patient samples.

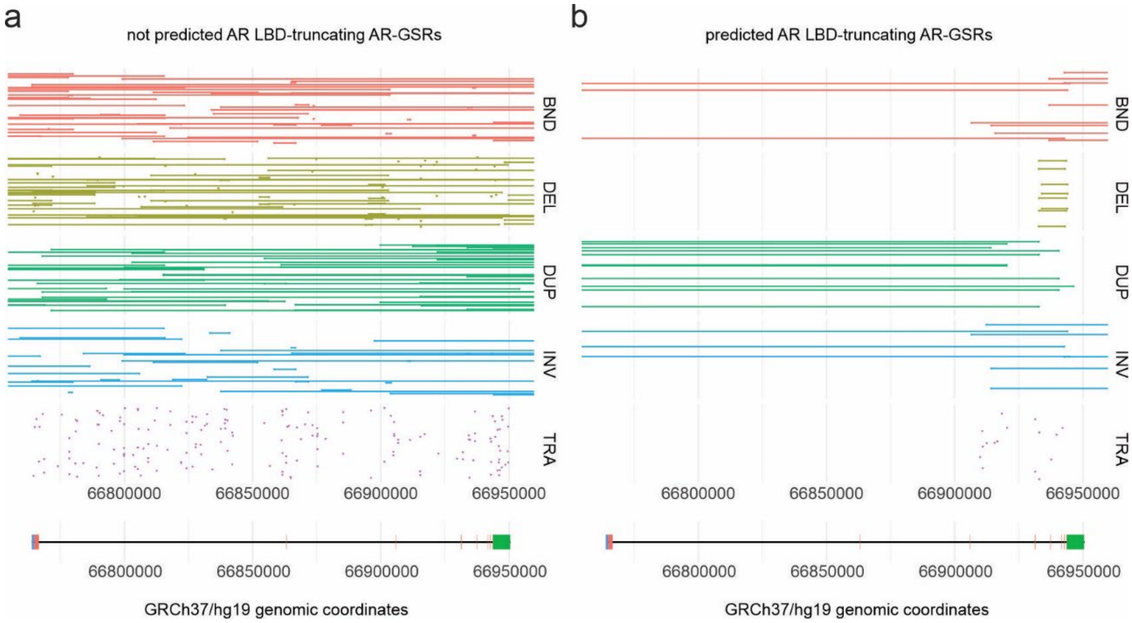

Supplementary Figure 3. Functional annotation of *AR*-GSRs. (a&b) Illustrations of breakpoint locations and genomic architectures for indicated structural variant classes (BND = breakend, DEL = deletion, DUP = duplication, INV = inversion, TRA = translocation). The *AR* gene is illustrated at the bottom. *AR*-GSRs are split into 2 groups based on whether they are (a) not predicted to truncate the AR ligand binding domain (LBD) or (B) likely to truncate the AR ligand binding domain.

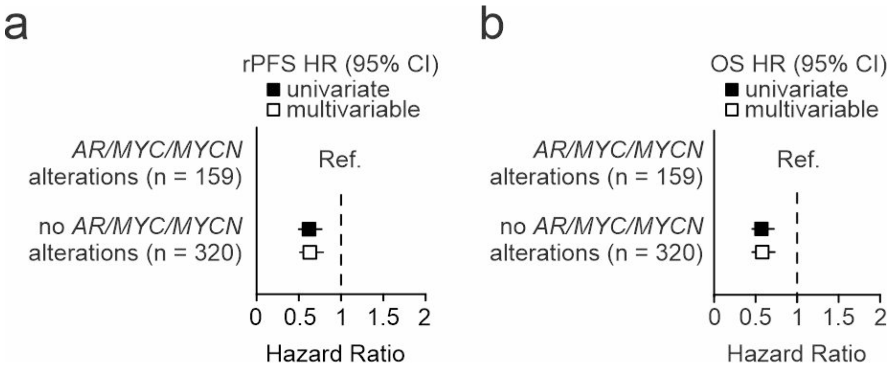

Supplementary Figure 4. Prognostic importance of *AR/MYC/MYCN* alterations in aneuploidy-low cfDNA specimens. (a&b) Forest plots illustrating hazard ratio (squares) and 95% confidence intervals (horizontal lines) for (a) radiographic progression (rPFS) and (b) death (OS) in ctDNA aneuploidy-low patients lacking pathogenic mutations but demonstrating alterations in *AR*, *MYC*, and/or *MYCN*. Multivariable analysis is adjusted for ctDNA aneuploidy fraction. ‘n’ refers to the number of patients.

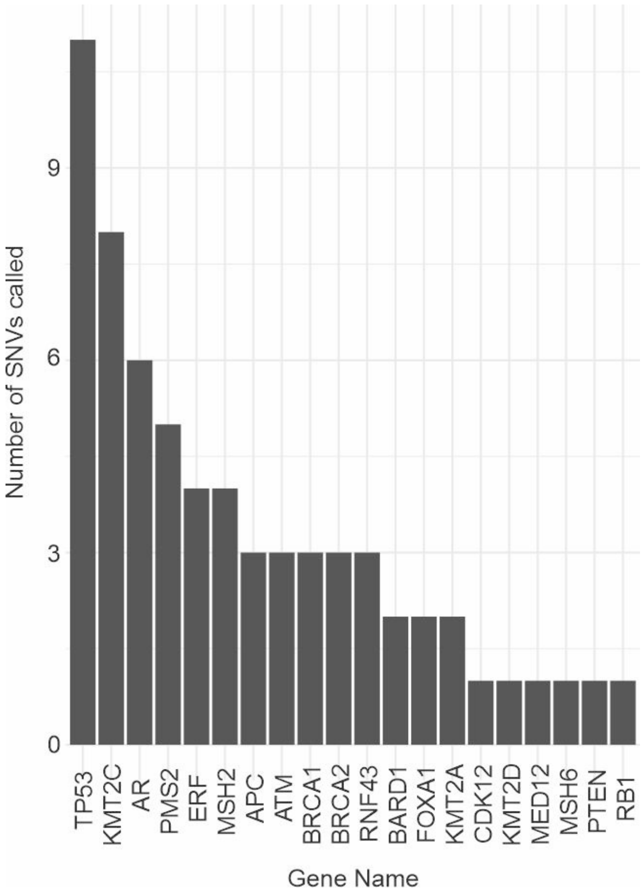

Supplementary Figure 5. ctDNA-defining mutations in ctDNA aneuploidy-low Group 2. Genes from the 39 samples in ctDNA aneuploidy-low Group 2 affected by a likely-somatic pathogenic mutation but lacking AR and/or AR enhancer copy gain, MYC and/or MYCN copy gain, or an AR-GSR.

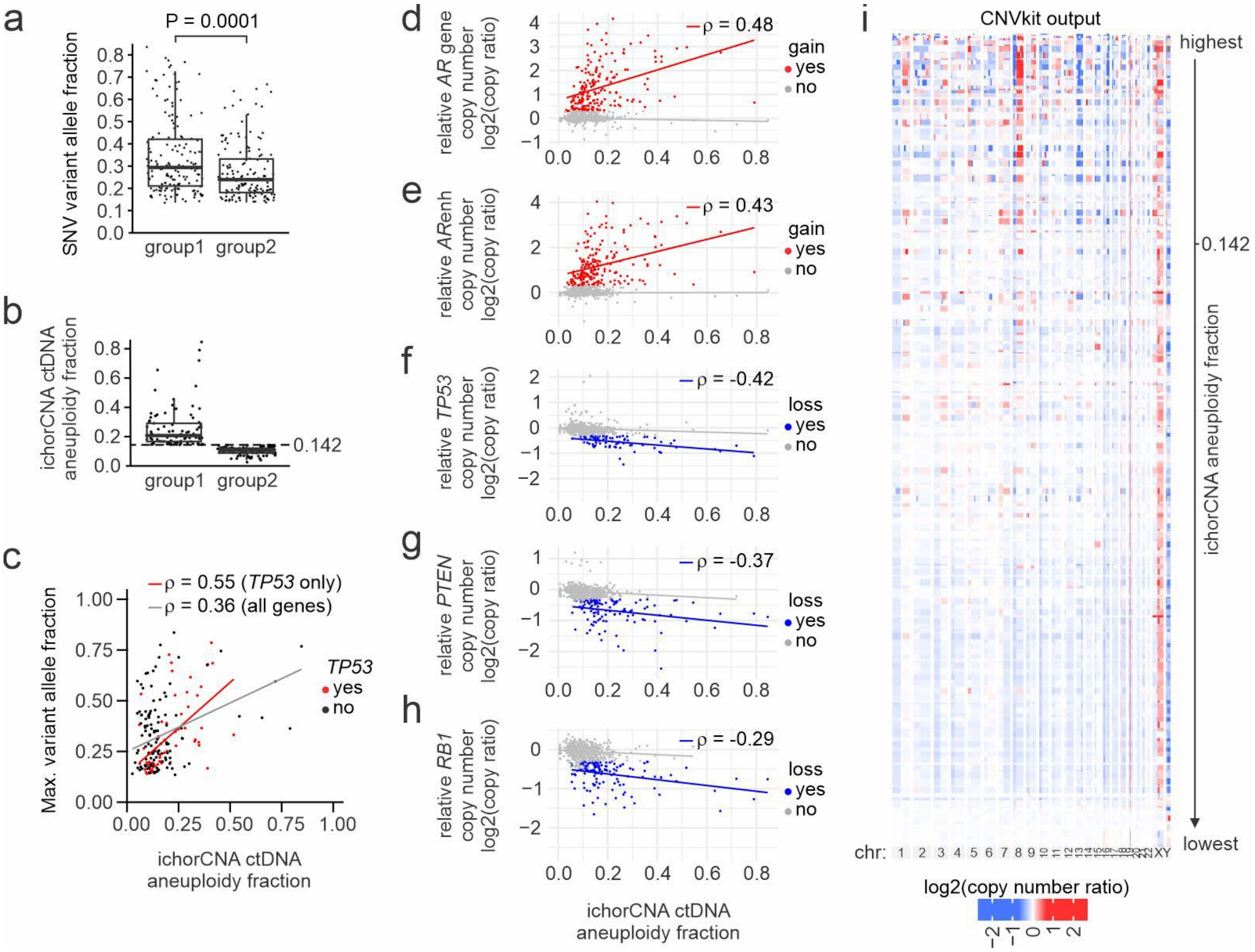

Supplementary Figure 6. Benchmarking ichorCNA aneuploidy fraction against alternative inputs for calculating ctDNA fraction. (a) Boxplot of variant allele fractions (VAFs) of pathogenic mutations detected in ctDNA-positive samples classified as ctDNA aneuploidy-high (Group 1) or ctDNA aneuploidy-low (Group 2). The p-value is determined from a Mann Whitney U-test. . Boxes represent median and interquartile range. Whiskers represent 1.5X interquartile range. **(b)** Boxplot of ichorCNA ctDNA aneuploidy fraction for samples as in (A). . Boxes represent median and interquartile range. Whiskers represent 1.5X interquartile range. **(c)** Scatterplot of ichorCNA ctDNA aneuploidy fraction vs. maximum variant allele fraction for n = 183 cfDNA specimens harboring a pathogenic mutation. The most frequently-mutated gene (*TP53*) is denoted by red dots. Trendlines and Pearson correlations coefficients are shown for all genes (gray line) or for *TP53* only (red line). **(d-h)** Scatterplot of ichorCNA ctDNA aneuploidy fraction vs. log2 copy ratios for (d) *AR*, (e) *AR* enhancer, (f) *TP53*, (g) *PTEN*, and (h) *RB1*. Pearson correlation coefficients (ρ) are shown for samples with gain (red) or loss (blue) only. **(i)** Copy number alterations in n = 776 cfDNA specimens derived from analysis by CNVkit. cfDNA samples are ordered top to bottom by decreasing ichorCNA ctDNA aneuploidy fraction.

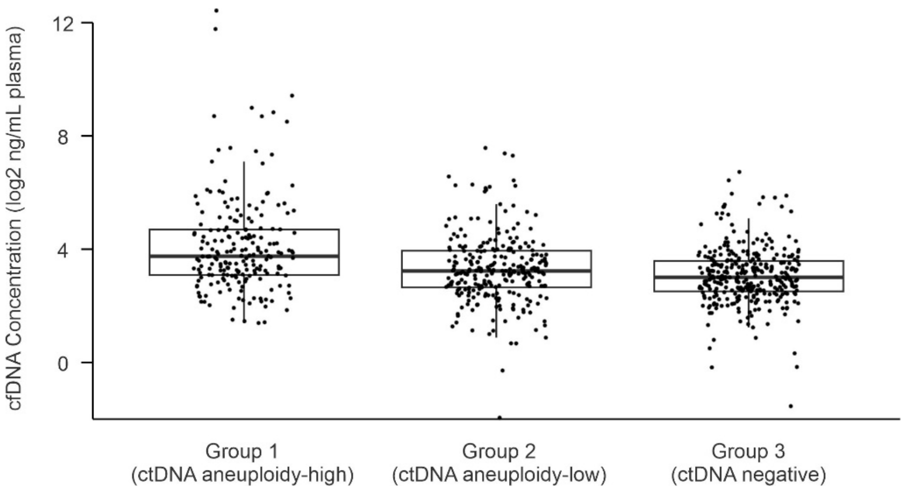

Supplementary Figure 7. cfDNA yields based on ctDNA group status. Boxplot of cfDNA yields for samples classified as belonging to ctDNA aneuploidy-high Group 1 (n = 200), ctDNA aneuploidy-low Group 2 (n = 256), or ctDNA-negative Group 3 (n = 320). Boxes represent median and interquartile range. Whiskers represent 1.5X interquartile range. 'n' refers to the number of patient samples.

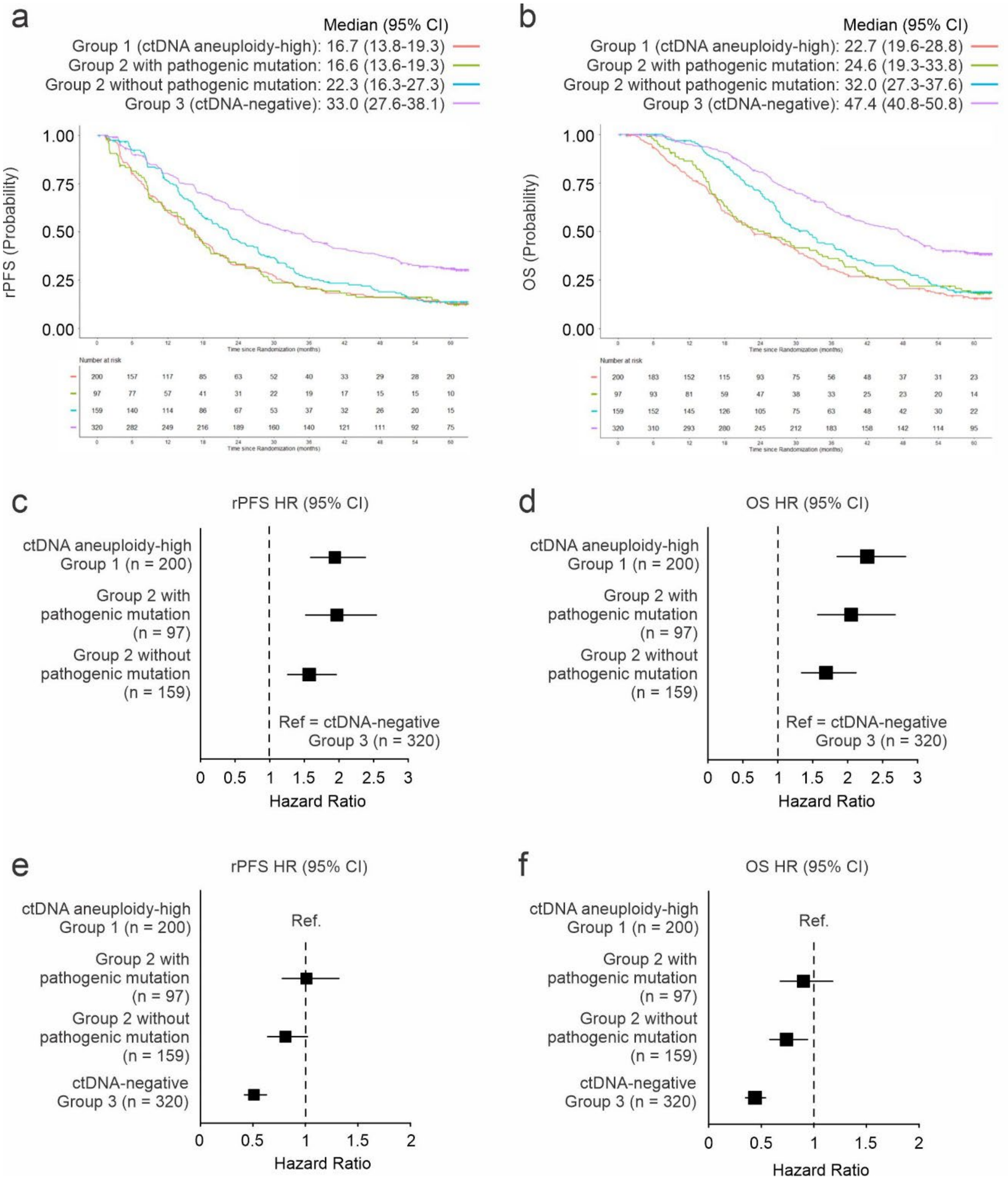

Supplementary Figure 8. Prognostic evaluation of patients in ctDNA aneuploidy-low Group 2 stratified by presence/absence of a pathogenic mutation. (a&b) Kaplan-Meier plots of (a) radiographic progression-free survival (rPFS) and (b) overall survival (OS) in ctDNA aneuploidy-positive and -low Groups 1-3. ctDNA-positive Group 2 patients are separated into subgroups containing (n = 97) or lacking (n = 159) a detectable pathogenic mutation. (**c&d**) Forest plots illustrating hazard ratio (squares) and 95% confidence intervals (horizontal lines) for (c) rPFS and (d) death (OS) in groups shown in a&b. Comparisons are relative to ctDNA-negative Group 3. 'n' refers to the number of patients. **(e&f)** Forest plots as in c&d, with hazard ratios relative to ctDNA-positive Group 1. 'n' refers to the number of patients.

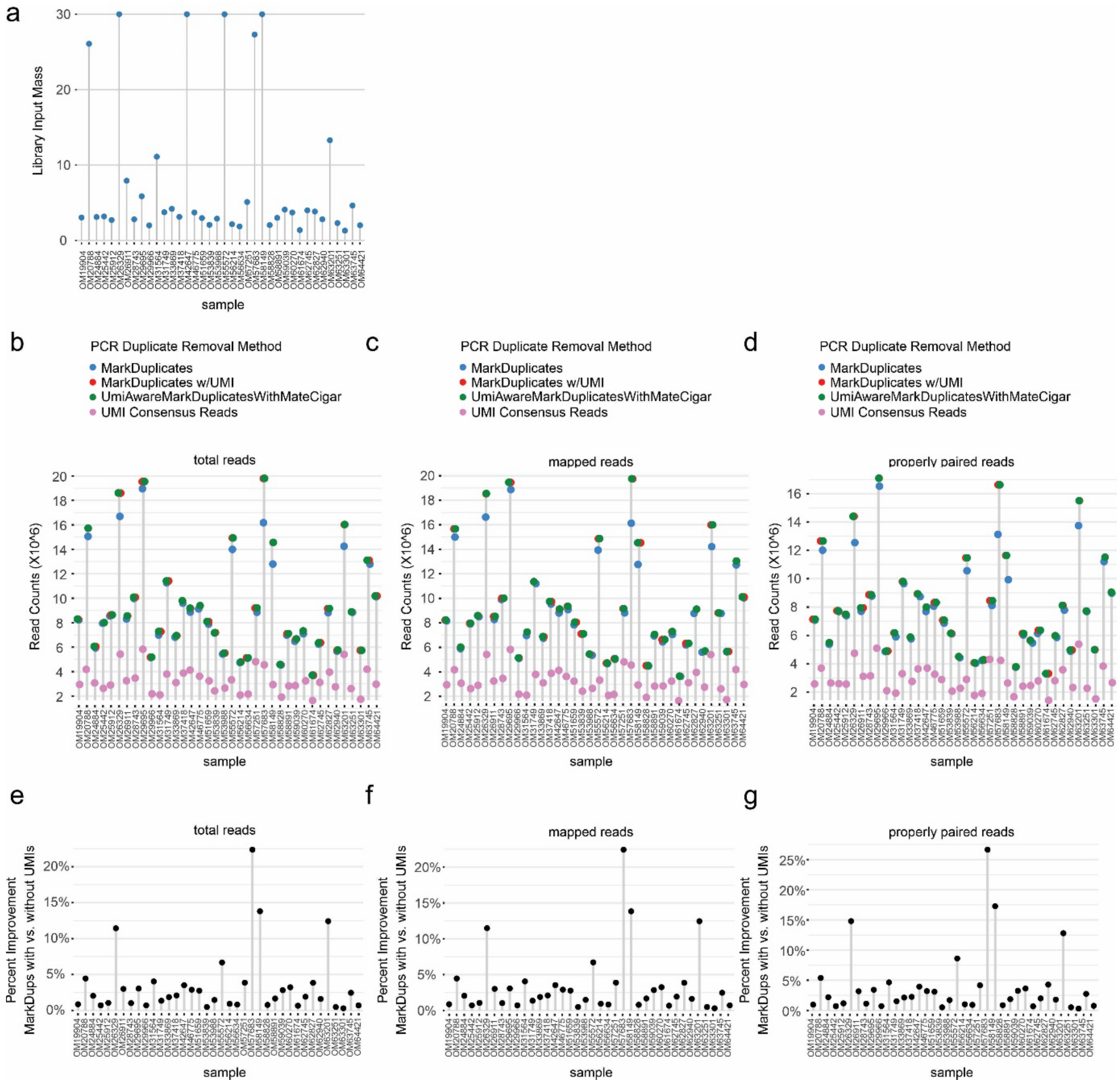

Supplementary Figure 9. PCR duplicate removal strategies. (a) Mass of DNA used as input for DNA-seq library preparation in a pilot DNA-seq study of 38 cfDNA samples. **(b)** Number of total DNA-seq reads remaining per sample after removal of PCR duplicates using Picard MarkDuplicates with default settings (blue), Picard MarkDuplicates with unique molecule index (UMI)-aware settings (red), Picard UmiAwareMarkDuplicatesWithMateCigar (green), or UMI Consensus Reads (pink). **(c)** Number of mapped DNA-seq reads remaining per sample after removal of PCR duplicates as in (b). **(d)** Number of mapped and properly-paired DNA-seq reads remaining per sample after removal of PCR duplicates as in (b). **(e)** Percent improvement in total DNA-seq reads remaining after removal of PCR duplicates using Picard MarkDuplicates with UMI-aware settings vs. Picard MarkDuplicates with default settings from (b). **(f)** Percent improvement in mapped DNA-seq reads remaining after removal of PCR duplicates using Picard MarkDuplicates with UMI-aware settings vs. Picard MarkDuplicates with default settings from (c). **(g)** Percent improvement in properly paired DNA-seq reads remaining after removal of PCR duplicates using Picard MarkDuplicates with UMI-aware settings vs. Picard MarkDuplicates with default settings from (d).
