# Supplementary material for: AR alterations inform circulating tumor DNA detection in metastatic castration resistant prostate cancer patients
Source: Nat Commun. 2024 Dec 11;15:10648. doi: 10.1038/s41467-024-54847-1 (PMC11634963; doi:10.1038/s41467-024-54847-1)
Supplement: Supplementary file 3 — Description of Additional Supplementary Files [file 41467_2024_54847_MOESM3_ESM.pdf]

Description of Additional Supplementary Files

Supplementary Data 1. Details of Agilent SureSelect Target Panel

Supplementary Data 2. cfDNA yield, tumor aneuploidy fraction, and ctDNA detection in A031201 cfDNA

Supplementary Data 3. Pathogenic mutations detected in A031201 ctDNA

Supplementary Data 4. DNA-seq coverage and log2 copy number ratios of targeted genes relative to control regions

Supplementary Data 5. Counts of ARGSRs detected in A031201 cfDNA

Supplementary Data 6. Breakpoint details of AR-GSRs detected in A031201 cfDNA
