# Supplementary material for: AR alterations inform circulating tumor DNA detection in metastatic castration resistant prostate cancer patients
Source: Nat Commun. 2024 Dec 11;15:10648. doi: 10.1038/s41467-024-54847-1 (PMC11634963; doi:10.1038/s41467-024-54847-1)
Supplement: Supplementary file 5 — Reporting Summary [file 41467_2024_54847_MOESM5_ESM.pdf]

nature portfolio

Corresponding author(s): Scott Dehm

Last updated by author(s): Oct 20, 2024

Reporting Summary

Nature Portfolio wishes to improve the reproducibility of the work that we publish. This form provides structure for consistency and transparency in reporting. For further information on Nature Portfolio policies, see our Editorial Policies and the Editorial Policy Checklist.

Statistics

For all statistical analyses, confirm that the following items are present in the figure legend, table legend, main text, or Methods section.

n/a	Confirmed	
☐	☒	The exact sample size (*n*) for each experimental group/condition, given as a discrete number and unit of measurement
☐	☒	A statement on whether measurements were taken from distinct samples or whether the same sample was measured repeatedly
☐	☒	The statistical test(s) used AND whether they are one- or two-sided<br>*Only common tests should be described solely by name; describe more complex techniques in the Methods section.*
☐	☒	A description of all covariates tested
☐	☒	A description of any assumptions or corrections, such as tests of normality and adjustment for multiple comparisons
☐	☒	A full description of the statistical parameters including central tendency (e.g. means) or other basic estimates (e.g. regression coefficient) AND variation (e.g. standard deviation) or associated estimates of uncertainty (e.g. confidence intervals)
☐	☒	For null hypothesis testing, the test statistic (e.g. *F*, *t*, *r*) with confidence intervals, effect sizes, degrees of freedom and *P* value noted<br>*Give P values as exact values whenever suitable.*
☒	☐	For Bayesian analysis, information on the choice of priors and Markov chain Monte Carlo settings
☒	☐	For hierarchical and complex designs, identification of the appropriate level for tests and full reporting of outcomes
☐	☒	Estimates of effect sizes (e.g. Cohen's *d*, Pearson's *r*), indicating how they were calculated

Our web collection on statistics for biologists contains articles on many of the points above.

Software and code

Policy information about availability of computer code

Data collection: no software was used for data collection

Data analysis:

No custom code was developed for data analysis. We used the following publicly-available code:

DNA-seq read mapping and duplicate removal:

Trimmomatic (v. 0.39) was used for removal of DNA-seq adapters and poor-quality bases.

umi-tools (v. 1.1.2) was used to identify, correct, and remove UMIs from the reads and place them into the BAM RX tag

bwa mem (v. 0.7.17) was used to map reads against the human reference genome (GRCh37/hg19)

samblaster (v. 0.1.24) was used to limit the number of split alignments for a read (max=2) and limit the number of non-overlapping base pairs between two alignments (min=20).

Picard tools (v. 2.25.5) MarkDuplicates was used for read duplicate marking and removal. This method was compared against Picard MarkDuplicates with the BARCODE_TAG option set to RX, Picard UmiAwareMarkDuplicatesWithMateCigar with the BARCODE_TAG option set to RX, FgBio (v. 1.3.0) functions SetMateInformation, GroupReadsByUmi (using RX tag), CallMolecularConsensusReads, FilterConsensusReads, and remapping deduplicated consensus reads with bwa mem.

samtools (v 1.12) flagstat was used to collect sequencing, mapping, and capture metrics

Picard tools (v. 2.25.5) functions (CollectInsertSizeMetrics, CollectAlignmentSummaryMetrics, CollectHSMetrics, CollectGCBiasMetrics) were used to collect sequencing, mapping, and capture metrics.

Estimation of ctDNA fraction:

ichorCNA R package (v.0.2.0) with R (v.4.2.0) were used to estimate the circulating tumor DNA (ctDNA) fraction using as input the duplicates-removed bam files that were filtered with bedtools (v. 2.3.0).

SNV/indel calling and filtering:

Freebayes (v.1.3.1) (and GATK MuTect2 (v.4.1.6.0) (35) were used to identify SNV/indel variants using the duplicates-removed BAM file as input.

rtg-tools (v.3.11) vcfeval function, with decompose and squash-ploidy parameters enabled, was used to compare filtered Freebayes and MuTect2 VCFs and intersecting calls were retained.

vcf2maf (v 1.6.21) was used to annotate variants, which depends on the Ensembl Variant Effect Predictor (v. 104.3), and OncoKB (v. 3.3.1).

Identification of copy number variants

mosdepth (v. 0.3.3) was used to calculate read depths using the duplicates-removed BAM file at every position targeted by the capture panel (i.e. gene exons or control regions) with no limit on the number of reads counted.

Identification of AR-GSRs

Manta (v.1.5.0), DELLY (v.0.8.1), SvABA (v.1.1.0), and LUMPY (v.0.3.0) were used to identify putative AR gene structural rearrangements (AR-GSRs). rtg-tools (v.3.11) svdecompose was used to convert VCFs from symbolic to breakend format from each caller. Variants from all intersecting and non-intersecting comparisons were imported into R (v. 4.2.0) where each was labeled, filtered, and counted using custom scripts.

Genome wide copy number alterations were inferred using CNVkit software (ver. 0.9.9). For each sample, the CNVkit coverage function was used with the inputs described above and the deduplicated BAM file to calculate read coverage levels across the targeted and off-target regions of the genome. This step required the hmmlearn software (ver. 0.2.7). For every sample, the inferred copy number ratios for each segment were plotted as a chromosomal heatmap using R and tidyverse functions.

For manuscripts utilizing custom algorithms or software that are central to the research but not yet described in published literature, software must be made available to editors and reviewers. We strongly encourage code deposition in a community repository (e.g. GitHub). See the Nature Portfolio guidelines for submitting code & software for further information.

Data

Policy information about availability of data

All manuscripts must include a data availability statement. This statement should provide the following information, where applicable:

- Accession codes, unique identifiers, or web links for publicly available datasets
- A description of any restrictions on data availability
- For clinical datasets or third party data, please ensure that the statement adheres to our policy

The raw DNA-seq data for this study are available in dbGaP under accession: phs003325.v1.p1 (https://www.ncbi.nlm.nih.gov/gap/). Processed DNA-seq data are in the Supplementary Data files S2-S6. Additional information and results related to the phase 3 A031201 trial can be found at clinicaltrials.gov under accession: NCT01949337 (https://www.clinicaltrials.gov/). The A031201 clinical trial protocol has been published previously31. The A031201 clinical data are available under restricted access from the NCI NCTN Data Archive (https://nctn-data-archive.nci.nih.gov/). All other data are all available in the main text, supplementary information or source data files. Source data is provided with this paper.

Research involving human participants, their data, or biological material

Policy information about studies with human participants or human data. See also policy information about sex, gender (identity/presentation), and sexual orientation and race, ethnicity and racism.

Reporting on sex and gender

Details of the patients, inclusion criteria, study endpoints, and specimens collected for the correlative analysis in this manuscript have been described previously (31).

31. Morris MJ, Heller G, Hillman DW, Bobek O, Ryan C, Antonarakis ES, et al. Randomized Phase III Study of Enzalutamide Compared With Enzalutamide Plus Abiraterone for Metastatic Castration-Resistant Prostate Cancer (Alliance A031201 Trial). J Clin Oncol 2023:JCO2202394.

This study did not consider sex or gender in the design.

Reporting on race, ethnicity, or

Details of the patients, inclusion criteria, study endpoints, and specimens collected for the correlative analysis have been described previously (31).

other socially relevant groupings	31. Morris MJ, Heller G, Hillman DW, Bobek O, Ryan C, Antonarakis ES, et al. Randomized Phase III Study of Enzalutamide Compared With Enzalutamide Plus Abiraterone for Metastatic Castration-Resistant Prostate Cancer (Alliance A031201 Trial). J Clin Oncol 2023:JCO2202394.
Population characteristics	Details of the patients, inclusion criteria, study endpoints, and specimens collected for the correlative analysis have been described previously (31).<br><br>31. Morris MJ, Heller G, Hillman DW, Bobek O, Ryan C, Antonarakis ES, et al. Randomized Phase III Study of Enzalutamide Compared With Enzalutamide Plus Abiraterone for Metastatic Castration-Resistant Prostate Cancer (Alliance A031201 Trial). J Clin Oncol 2023:JCO2202394.
Recruitment	Details of the patients, inclusion criteria, study endpoints, and specimens collected for the correlative analysis have been described previously (31).<br><br>31. Morris MJ, Heller G, Hillman DW, Bobek O, Ryan C, Antonarakis ES, et al. Randomized Phase III Study of Enzalutamide Compared With Enzalutamide Plus Abiraterone for Metastatic Castration-Resistant Prostate Cancer (Alliance A031201 Trial). J Clin Oncol 2023:JCO2202394.
Ethics oversight	Details of the patients, inclusion criteria, study endpoints, and specimens collected for the correlative analysis have been described previously (31). This study was approved by the University of Minnesota Institutional Review Board on 09/29/2020 (STUDY00010929) and the Duke University Institutional Review Board on 09/23/2020 (Pro00106740). Approval was received by the NCTN for correlative science proposal CSC0159 on June 16, 2021<br><br>31. Morris MJ, Heller G, Hillman DW, Bobek O, Ryan C, Antonarakis ES, et al. Randomized Phase III Study of Enzalutamide Compared With Enzalutamide Plus Abiraterone for Metastatic Castration-Resistant Prostate Cancer (Alliance A031201 Trial). J Clin Oncol 2023:JCO2202394.

Note that full information on the approval of the study protocol must also be provided in the manuscript.

Field-specific reporting

Please select the one below that is the best fit for your research. If you are not sure, read the appropriate sections before making your selection.

☒ Life sciences ☐ Behavioural & social sciences ☐ Ecological, evolutionary & environmental sciences

For a reference copy of the document with all sections, see nature.com/documents/nr-reporting-summary-flat.pdf

Life sciences study design

All studies must disclose on these points even when the disclosure is negative.

Sample size	Approval was received by the NCTN for correlative science proposal CSC0159 on June 16, 2021, enabling 2 mL of banked pre-treatment EDTA plasma specimens collected from patients at baseline to be shipped on dry ice from the Alliance biorepository at Ohio State University to the University of Minnesota, only for those patients where at least 2 mL of plasma was available. This ensured that banked plasma specimens from A031201 would not be exhausted by this study. Of the 1,311 patients enrolled in the A031201 trial, 776 samples were available for detailed analysis in this study. The CONSORT diagram in Figure 1A describes all patients that were included/excluded from the study, with rationale.
Data exclusions	The CONSORT diagram in Figure 1A describes all patients that were included/excluded from the study, with rationale.
Replication	There was no replication of experiments in this study. Each patient plasma sample was analyzed by the targeted DNA-sequencing assay one time.
Randomization	The Alliance A031201 trial was a randomized phase 3 clinical trial wherein men with previously untreated mCRPC and progressive metastatic disease despite ongoing androgen deprivation therapy (ADT) were treated with standard of care doses of enzalutamide 160 mg/d with or without abiraterone acetate and prednisone (1:1 randomization, open label) until clinical or radiographic progression and patients were followed long-term for mortality.
Blinding	Investigators at Duke University performed the statistical analyses to evaluate the prospective hypotheses that detection of AR-GSRs in ctDNA would be associated with worse overall survival (OS), and that ctDNA positive patients would have adverse baseline clinical features, worse radiographic progression free survival (rPFS), and worse OS. Investigators at the University of Minnesota performed cfDNA-sequencing and data analysis to identify ctDNA-positive patient groups, and were blinded to clinical data.

Reporting for specific materials, systems and methods

We require information from authors about some types of materials, experimental systems and methods used in many studies. Here, indicate whether each material, system or method listed is relevant to your study. If you are not sure if a list item applies to your research, read the appropriate section before selecting a response.

Materials & experimental systems

n/a	Involved in the study
☒	☐ Antibodies
☒	☐ Eukaryotic cell lines
☒	☐ Palaeontology and archaeology
☒	☐ Animals and other organisms
☐	☒ Clinical data
☒	☐ Dual use research of concern
☒	☐ Plants

Methods

n/a	Involved in the study
☒	☐ ChIP-seq
☒	☐ Flow cytometry
☒	☐ MRI-based neuroimaging

Clinical data

Policy information about clinical studies

All manuscripts should comply with the ICMJE guidelines for publication of clinical research and a completed CONSORT checklist must be included with all submissions.

Clinical trial registration

Alliance A031201 (Clinicaltrials.gov: NCT01949337)

Study protocol

The study protocol was provided with a previous publication:

31. Morris MJ, Heller G, Hillman DW, Bobek O, Ryan C, Antonarakis ES, et al. Randomized Phase III Study of Enzalutamide Compared With Enzalutamide Plus Abiraterone for Metastatic Castration-Resistant Prostate Cancer (Alliance A031201 Trial). J Clin Oncol 2023:JCO2202394.

Data collection

Details of the patients, inclusion criteria, study endpoints, and specimens collected for the correlative analysis have been described previously (31).

31. Morris MJ, Heller G, Hillman DW, Bobek O, Ryan C, Antonarakis ES, et al. Randomized Phase III Study of Enzalutamide Compared With Enzalutamide Plus Abiraterone for Metastatic Castration-Resistant Prostate Cancer (Alliance A031201 Trial). J Clin Oncol 2023:JCO2202394.

The A031201 clinical data are available under restricted access from the NCI NCTN Data Archive (https://nctn-data-archive.nci.nih.gov/).

Outcomes

Details of the patients, inclusion criteria, study endpoints, and specimens collected for the correlative analysis have been described previously (31).

31. Morris MJ, Heller G, Hillman DW, Bobek O, Ryan C, Antonarakis ES, et al. Randomized Phase III Study of Enzalutamide Compared With Enzalutamide Plus Abiraterone for Metastatic Castration-Resistant Prostate Cancer (Alliance A031201 Trial). J Clin Oncol 2023:JCO2202394.

The A031201 clinical data are available under restricted access from the NCI NCTN Data Archive (https://nctn-data-archive.nci.nih.gov/).

Plants

Seed stocks

Report on the source of all seed stocks or other plant material used. If applicable, state the seed stock centre and catalogue number. If plant specimens were collected from the field, describe the collection location, date and sampling procedures.

Novel plant genotypes

Describe the methods by which all novel plant genotypes were produced. This includes those generated by transgenic approaches, gene editing, chemical/radiation-based mutagenesis and hybridization. For transgenic lines, describe the transformation method, the number of independent lines analyzed and the generation upon which experiments were performed. For gene-edited lines, describe the editor used, the endogenous sequence targeted for editing, the targeting guide RNA sequence (if applicable) and how the editor was applied.

Authentication

Describe any authentication procedures for each seed stock used or novel genotype generated. Describe any experiments used to assess the effect of a mutation and, where applicable, how potential secondary effects (e.g. second site T-DNA insertions, mosiacism, off-target gene editing) were examined.
